# Comparison of the Effects of Different Palatal Morphology on Maxillary Expansion via RME and MSE: A Finite Element Analysis

**DOI:** 10.1002/cre2.70005

**Published:** 2024-09-19

**Authors:** Yaohui Pan, Wenjing Peng, Yanyu Wang

**Affiliations:** ^1^ Department of Orthodontics Wuxi Stomatological Hospital Wuxi Jiangsu China; ^2^ Hospital of Stomatology Xuzhou Medical University Xuzhou Jiangsu China; ^3^ Foshan Stomatological Hospital School of Stomatology and Medicine, Foshan University Foshan China

**Keywords:** finite element analysis, maxillary expansion, MSE, palatal morphology, RME

## Abstract

**Objectives:**

This study aims to compare and analyze the biomechanical effect and the displacement trend of RME and MSE on the maxillofacial complex under different palatal shapes by using finite element analysis.

**Methods:**

The three‐dimensional model of maxillofacial complex was obtained from a computed tomography image of a person with a normal palate. Then, we modified the shape of the palate to obtain the model with a high palate. Additionally, two expander devices were considered. MSE and RME were created and four models were made: Model 1: Normal‐palate craniomaxillofacial complex with RME expander; Model 2: Normal‐palate craniomaxillofacial complex with MSE expander; Model 3: High‐palate craniomaxillofacial complex with RME expander; Model 4: High‐palate craniomaxillofacial complex with MSE expander. Then, lateral forced displacement was applied and the analysis results were obtained.

**Results:**

The lateral displacement of the palatal suture of Model 3 is greater than that of Model 1, and the maxilla has more rotation. The crown/root ratio of Model 1 is significantly greater than that of the other three groups. Compared with Model 1, Model 3 has greater stress concentration in the superstructure of the craniomaxillofacial complex. Both of them have greater stress in the anchorage area than Model 2 and Model 4.

**Conclusion:**

Different shapes of the palate interfere with the effects of RME and MSE, and its influence on the stress distribution and displacement of the craniomaxillary complex when using RME is greater than MSE. The lateral displacement of the palatal suture of MSE is significantly larger than that of RME. It is more prone to tipping movement of the anchor teeth using RME under normal palate, and MSE may manage the vertical control better due to the smaller crown/root ratio than RME and intrusive movement of molars.

## 1 Introduction

Maxillary transverse deficiency (MTD) is a common clinical problem. Inadequate maxillary width has been reported in 9.4% of the population and nearly 30% of adult patients (Brunelle, Bhat, and Lipton 1996). MTD often leads to severe malocclusion, such as crowding and crossbite, which not only affect occlusal function and esthetics, but also may cause functional problems such as upper airway narrowing, increased nasal airway resistance, and altered tongue position (McNamara et al. 2015). Clinical patients with high‐narrow palate, narrowing of the dental arch, and skeletal class III patients with insufficient maxillary development usually require maxillary expansion treatment. Maxillary rapid expansion has been widely used in the treatment of insufficient maxillary width, and its expansion effect comes mainly from three parts: Expansion of the mid‐palatal suture, expansion of the alveolar bone, and tipping movement of the teeth (Liu, Xu, and Zou 2015).

However, many undesirable side effects of conventional RME have been identified, such as dentoalveolar tipping, drop of the palatal cusp, increase of the Wilson curve, root resorption, decrease of the level of alveolar bone, gingival recession, and periodontal dehiscence (Garib et al. 2005; Lemos Rinaldi et al. 2018; Baysal et al. 2013; Lo Giudice et al. 2018). To increase the orthopedic effect and reduce the side effects of traditional RME, surgically assisted rapid maxillary expansion (SARME) and various bone‐borne anchorage devices have been introduced and have shown clinical success (Lee et al. 2010; Gunyuz Toklu, Germec‐Cakan, and Tozlu 2015; Lin et al. 2015; Asscherickx et al. 2016). However, SARME is an invasive process with greater trauma and pain. Most of the currently available expanders are hybrid and are composed of both miniscrews and tooth‐borne parts. MARPE is either a tooth‐bone‐borne or a solely bone‐borne RPE device with a rigid element that connects to miniscrews inserted into the palate, delivering the expansion force directly to the basal bone of the maxilla (Lee et al. 2010). It was designed to maximize skeletal effects and to minimize dentoalveolar effects of expansion, based on the findings of previous histological studies revealing that the midpalatal suture does not fully ossify in humans even at an elderly age, possibly due to the constant mechanical stress that it undergoes (N'Guyen, Ayral, and Vacher 2008; Poorsattar Bejeh Mir et al. 2016). Maxillary skeletal expander (MSE) is a particular type of MARPE that can achieve orthopedic expansion of the palate by bicortical mini‐implant anchorage even in adults (Lee, Moon, and Hong 2017).

In clinical practice, patients with mouth breathing often have narrow dental arches, high arched palate, and protruding upper front teeth, while patients with skeletal type III often suffer from underdevelopment of the maxilla and overdevelopment of the mandible. Although their palate morphology differs, they often both have imbalances in the width of the upper and lower dental arches and require arch expansion. There are differences in the morphology of the palate in patients, which makes differences in the location of the expander and the effect of the maxillary expansion, and there is currently a lack of research on the influence and mechanism of the palatal morphology on the effect of expansion. Although the literature has reported the influence of palatal depth on the mechanical effect and displacement trend of the maxillary body and the dentition during the expansion (Matsuyama et al. 2015), it only established the maxilla model rather than a three‐dimensional model of the craniomaxillofacial complex, so its simulation effect is limited.

In this study, the palatal index (PI) was used to objectively evaluate the shape of the palate, avoiding the influence of subjective factors and visual errors (Paul and Nanda 1973). The palatal morphology is classified by calculating the ratio of the palatal height to the palatal width between the maxillary first and second premolars. Based on this index, a craniomaxillary complex model with normal palatine and high‐arched palatine was established.

Mechanical distribution properties of the craniomaxillary complex resulting from maxillary expansion cannot be obtained by using traditional cephalometric appraisals. Finite element analysis provides a proven method that can achieve structural simulation and mechanical analysis and has been used extensively in research on maxillary expansion (Lee, Moon, and Hong 2017). Finite element analysis can replace complex structures with a finite number of elements with simple geometric shapes, and play an essential role in the field of medical biomechanics (Panagiotopoulou 2009). Therefore, this study aims to simulate the mechanical effects of RME and MSE on craniomaxillofacial complex and bone sutures and analyze the displacement of the maxilla, mid‐palatal suture, and dentition using three‐dimensional finite element models of different palate morphologies. The results of the present study can provide a certain theoretical basis for the selection, design, and practical application of RME and MSE in clinical practice.

## 2 Materials and Methods

A patient with normal palatal morphology (PI = 36%) was selected as the subject for this study. The subject was approved by the hospital's ethical committee and the patient has signed an informed consent. The finite element model of the craniomaxillofacial complex was generated using volumetric data from the CBCT scan (slice thickness, 0.3 mm) using Mimics software (version 20.0; Materialise, Belgium). First, determine the threshold of teeth and bone tissue, reduce noise, extract the corresponding image data as accurately as possible, segment it with other structures such as soft tissue, and establish a preliminary craniomaxillary complex mask. Then, use region growing to concentrate pixels with similar properties to eliminate noise, soft tissue, and artifacts. Use mask editing to erase unnecessary parts, leaving only the craniomaxillary complex, fill in needed but discontinuous structures, and finally establish an accurate three‐dimensional structure of the craniomaxillary complex. The reconstructed model was exported to 3‐matic Research (version 12.0; Materialise, Belgium) where frontomaxillary, zygomaticomaxillary, zygomaticotemporal, pterygopalatal, and mid‐palatal sutures were marked and the thickness of the sutures was considered to be 0.5 mm (Fricke‐Zech et al. 2012); then, the part of the suture surface was removed based on Boolean operation and a model structure similar to the physiological structure was obtained. The completed bone suture model was exported in stl format and then the stl file was imported into Geomagic Studio 2014 (Geomagic, American) to construct the normal‐palate entity model and perform noise reduction and model trimming. The periodontal ligament (PDL) was modeled on the root shape with an average thickness of 0.25 mm.

**Figure 1:**
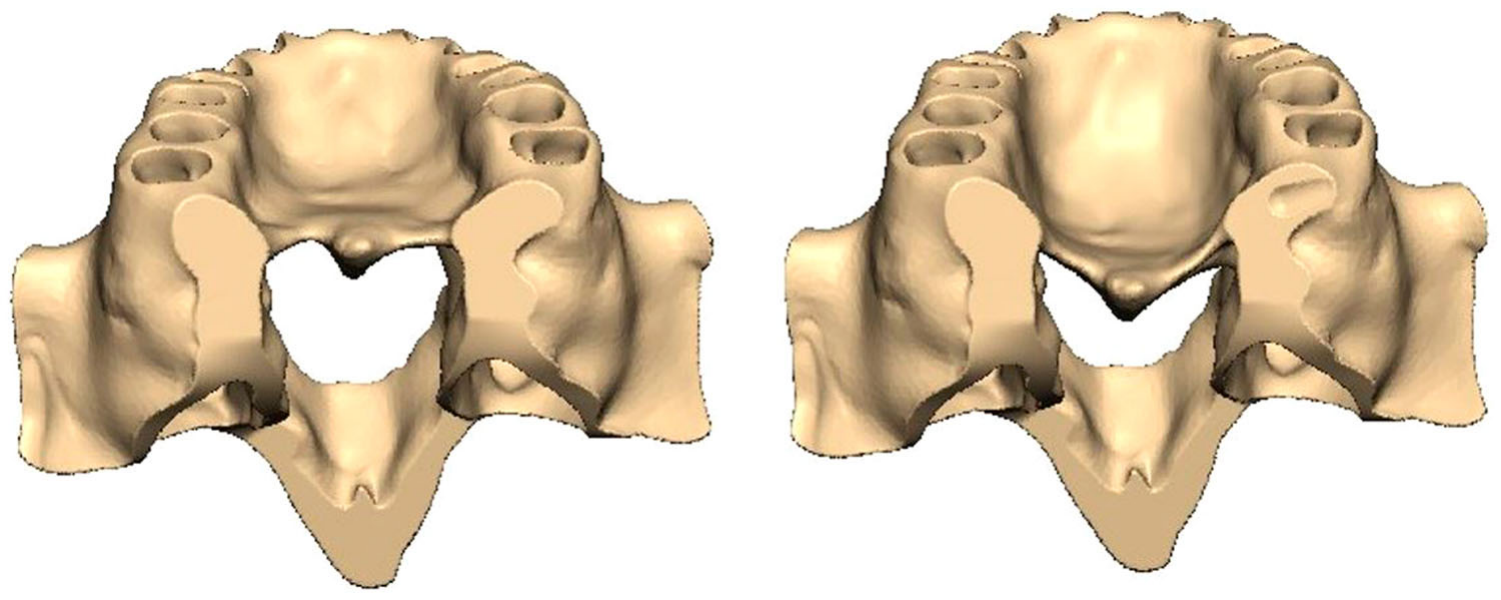
Comparison of palatal morphology in two models.

In this study, PI is used to objectively describe the palate morphology. Redman, Shapiro, and Gorlin (1966) first described the PI for palatal measurements and established standards of palatal dimension and shape to compare the reportedly malformed palates. The index indicates the relative height or narrowness of a palate and has been used in some studies on palatal morphology (Aluru et al. 2023). PI is the ratio of the palatal height to the palatal width between the first premolar and the second premolar, and it can be regarded as high‐narrow palate when the PI > 41% (Howell 1981; Perkiömäki and Alvesalo 2008). Then, the high‐palate model was obtained by the reconstruction of the palatal morphology of the craniomaxillofacial complex using Geomagic Studio 2014 with PI = 50% (Figure 1).

In the ANSYS Workbench software (version 19.0; ANSYS, American), corresponding Hyrax and MSE entity models based on physical dimensions were established on the normal‐palate and high‐palate craniomaxillofacial models, respectively. The four implants of MSE were constructed as cylindrical structures (length, 11.0 mm; diameter, 1.5 mm) and were implanted perpendicular to the bone surface at the outer 3 mm of the mid‐palatal suture (Park et al. 2017). The Hyrax arms are connected to the lingual surface of the first premolars and the first molars, respectively, and the MSE arms are connected to the lingual surface of the first molars.

**Figure 2:**
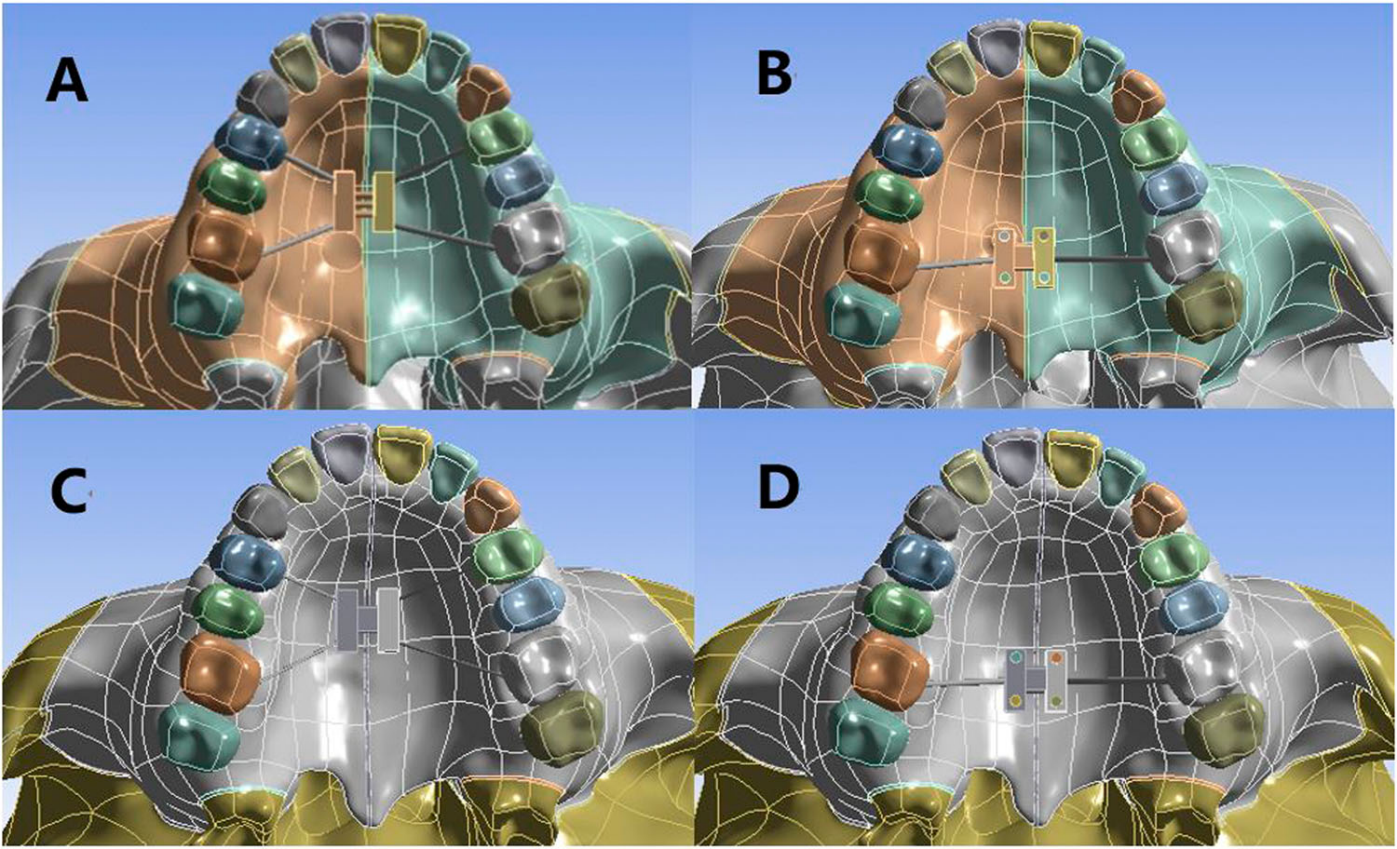
Location of RME and MSE ([A]: normal palate + RME, [B]: normal palate + MSE, [C]: high palate + RME, [D]: high palate + MSE).

Four models were made by assembling the craniomaxillofacial complexes and the expanders in Ansys 19.0 (Figure 2).

- Model 1: Normal‐palate craniomaxillofacial complex with RME expander
- Model 2: Normal‐palate craniomaxillofacial complex with MSE expander
- Model 3: High‐palate craniomaxillofacial complex with RME expander
- Model 4: High‐palate craniomaxillofacial complex with MSE expander

**Figure 3:**
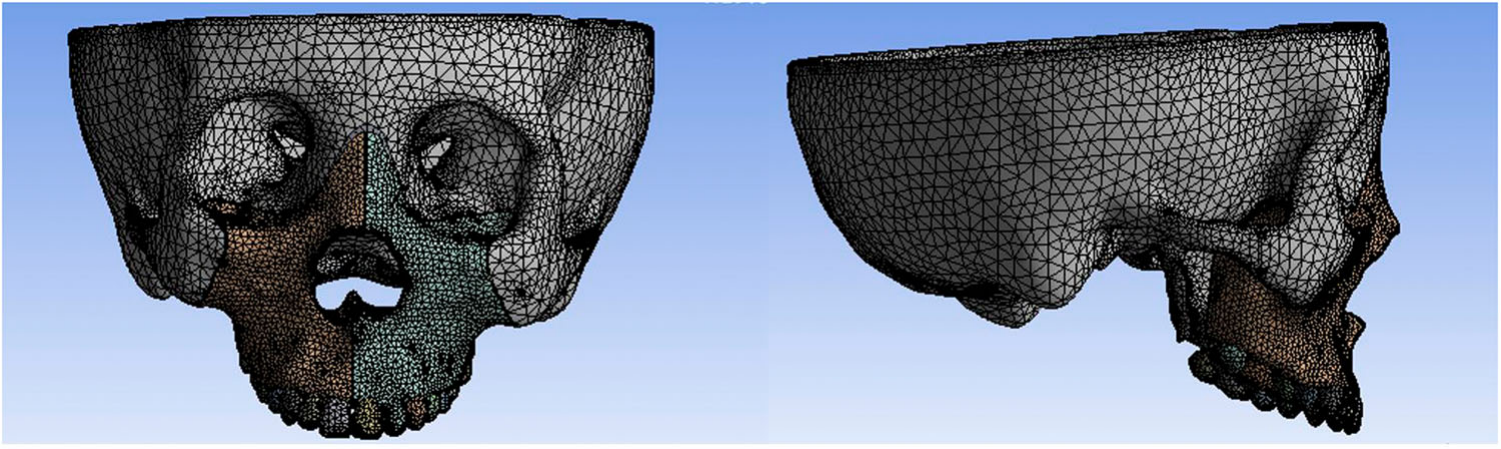
Establishment of mesh models in Ansys 19.0.

Four‐noded tetrahedral elements were used for volumetric mesh generation using Ansys 19.0 (Figure 3). The maxilla, alveolar bone, dentition, and bone sutures were sectioned into 1 mm tetrahedrons aimed at increasing the accuracy of models. Other parts of the craniomaxillofacial complex were sectioned into 5 mm tetrahedrons (Meng et al. 2022; Priyadarshini et al. 2017). The modulus of elasticity and Poisson's ratio for cortical bone, cancellous bone, bone sutures, teeth, implants, and expanders were defined (Table 1). The connection relationship among different structures was set (Table 2). The three‐dimensional coordinates were x (horizontal plane), y (sagittal plane), and z (vertical plane) and the positive values were set rightward, backward, and upward. Model material properties were defined as homogeneous, continuous, and isotropic based on previous research, and many studies have shown the accuracy of the finite element analysis which simulates the mechanical behavior of complex biologic structures (Park et al. 2017; Bezerra et al. 2013). The expanders were activated by applying 0.5 mm of transverse forced displacement along the *X*‐axis in the four models, 0.25 mm on each side.

**Table 1:** Material properties.

Component	Young's modulus (MPa)	Poisson's ratio
Cortical bone	1.37 × 10^4^	0.3
Cancellous bone	7.9 × 10^3^	0.3
Teeth	2.07 × 10^4^	0.3
Bone sutures	0.68	0.4
Expanders	2.0 × 10^5^	0.3
Implants	1.1 × 10^5^	0.3

**Table 2:** Connection relationship.

Structure	Connection relationship
Maxilla and bone sutures	Bonded
Palate and implants	Bonded
Expander and implants	Bonded
Expander arms and anchor teeth	Bonded
Maxilla and other bones	No separation
Roots and alveolar bone	No separation

**Figure 4:**
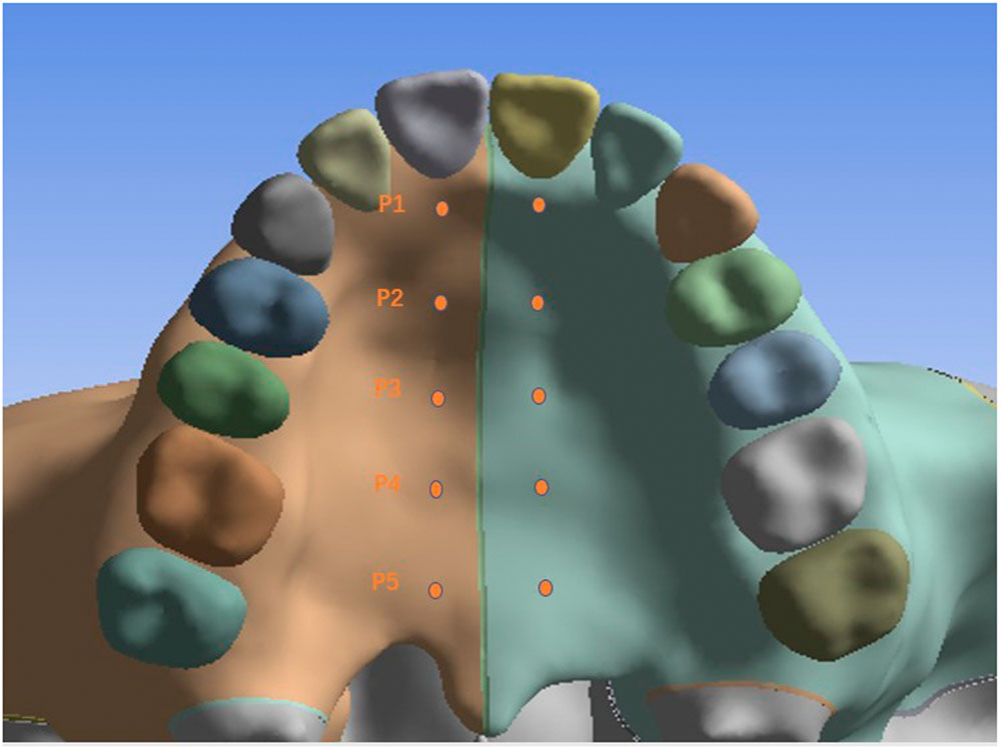
Palatal landmarks.

**Figure 5:**
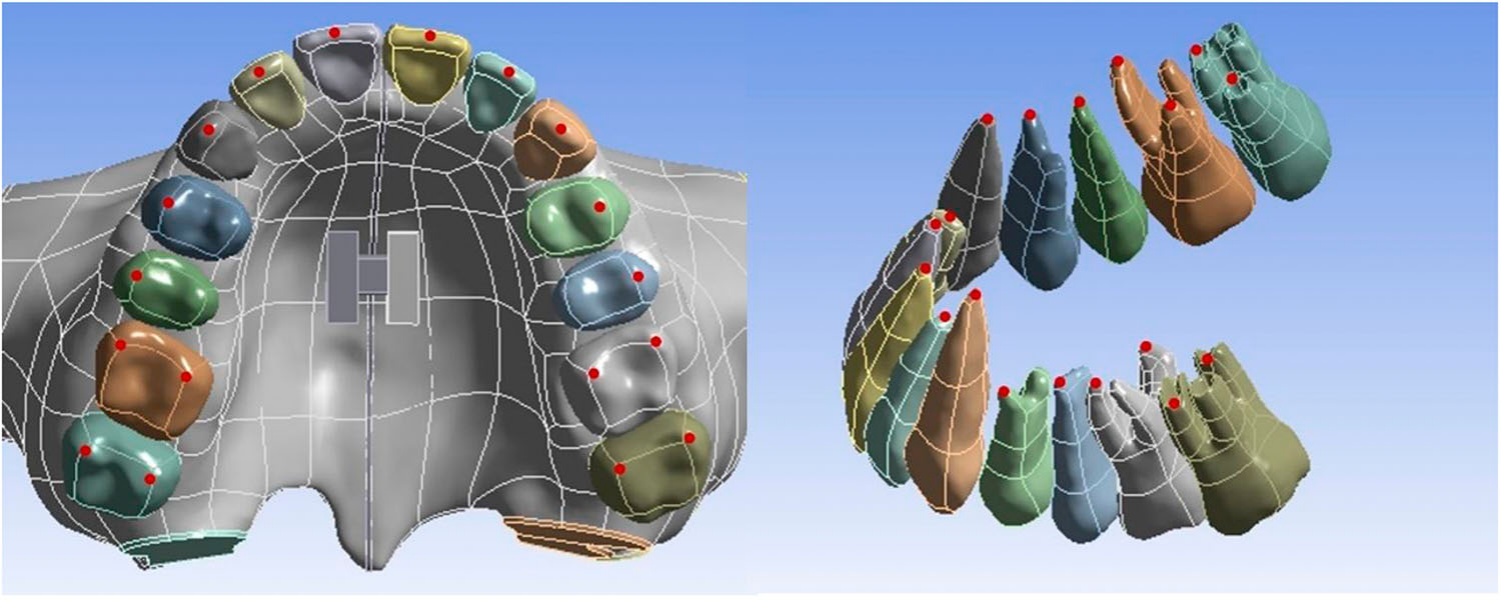
Occlusal and radicular dental landmarks.

Five landmarks of palate were measured to evaluate the displacement of mid‐palatal suture, which are on the level of the cusp of the canine, the buccal cusp of the first premolar, the buccal cusp of the second premolar, the mesiopalatal cusp of the first molar, and the mesiopalatal cusp of the second molar, respectively. The points are also on the parallel line of the mid‐palatal suture at the midpoint of the lingual cervical margin of the central incisor (Figure 4). Several landmarks of teeth were measured to evaluate the three‐dimensional displacement of dentition. The occlusal landmarks are the midpoint of the incisal edge, the cusp of canine, the buccal cusp of premolar, and the mesiobuccal and mesiopalatal cusp of molar. The radicular landmarks are the apex of the anterior teeth, the apex of the buccal root of the premolar, the apex of the mesiobuccal, and the palatal root of the molar (Figure 5). These landmarks could be visualized clearly and located accurately in three‐dimensional finite element models of cranial‐maxillary complex and ensure measurement data validation and accuracy (Park et al. 2017; Eom et al. 2018).

## 3 Results

### 3.1 Von Mises Stress Distribution on Craniofacial Complex

**Figure 6:**
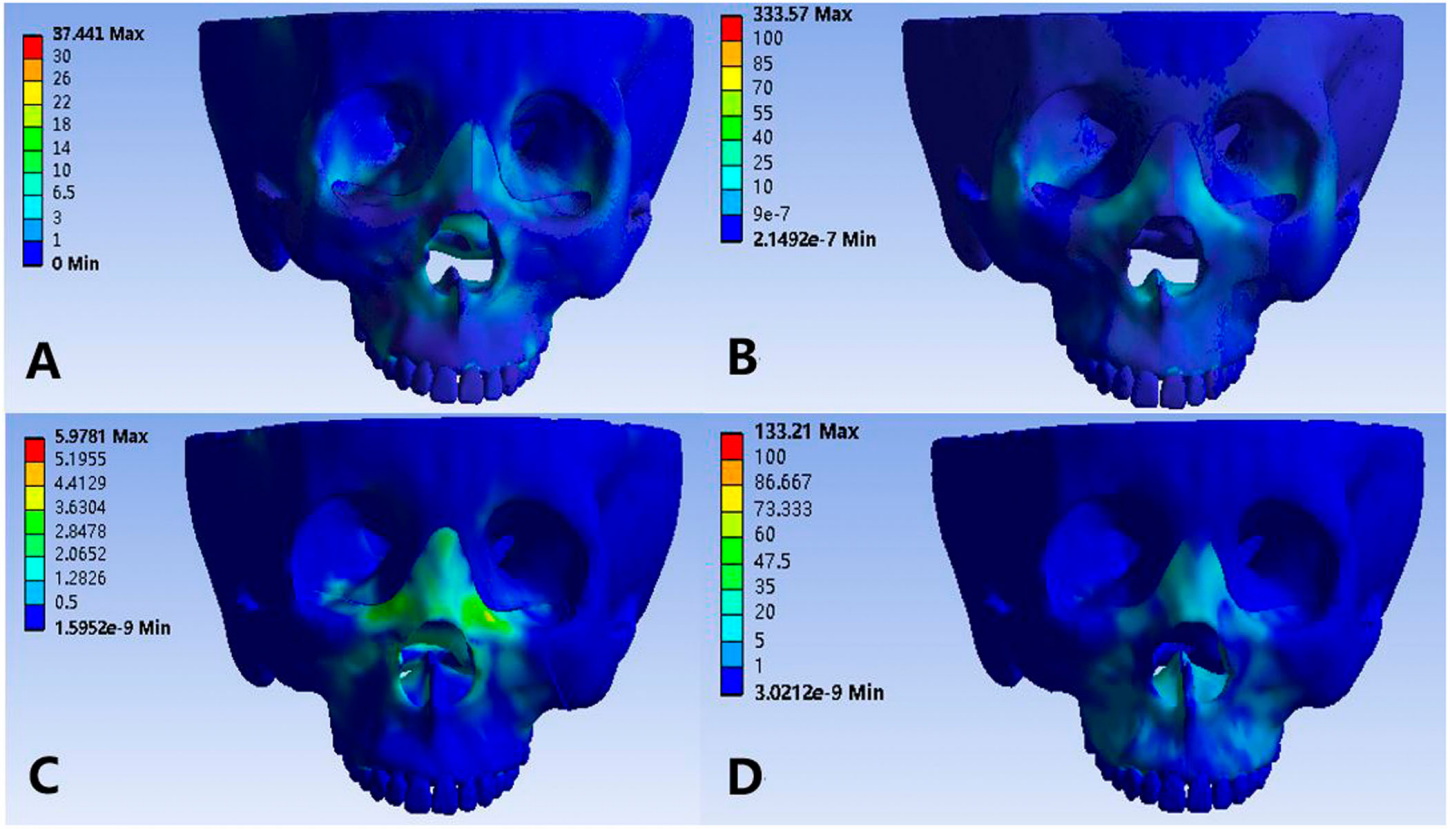
Von Mises stress comparison on craniofacial complex (frontal view). (A) Normal palate + RME, (B) normal palate + MSE, (C) high palate + RME, and (D) high palate + MSE.

**Figure 7:**
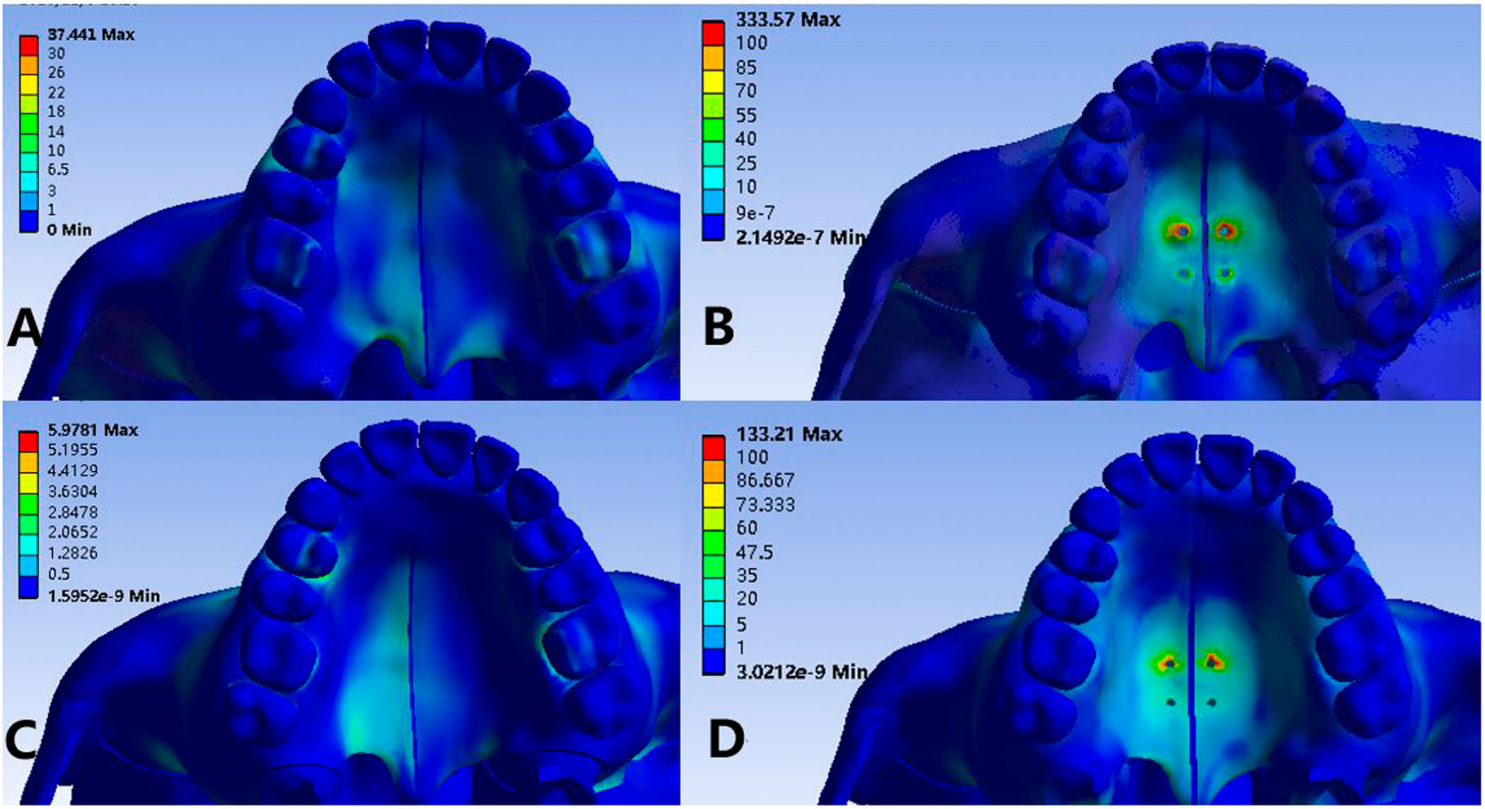
Von Mises stress comparison on craniofacial complex (occlusal view). (A) Normal palate + RME, (B) normal palate + MSE, (C) high palate + RME, and (D) high palate + MSE.

Through the comparative analysis of the equivalent stress nephograms of the four models (Figures 6 and 7), it is found that Model 3 has the smallest equivalent stress on the craniomaxilla, but the frontal process of the maxilla, the medial orbital wall, the orbital floor, and buccolingual alveolar region of the anchoring teeth have obvious stress concentration. The stress on the craniomaxillary complex in Model 1 is slightly greater than that in Model 3. The maximum equivalent stress is located in the buccal alveolar process of the maxillary first premolar, in addition, the periphery of the piriform foramen, the frontal process of the maxilla, and the medial orbital wall also have stress distribution. The stress on Model 2 and Model 4 is significantly greater than that on Model 1 and Model 3, and the stress is concentrated in the area around the implant nails, and the two implant nails in the front are stressed more than the rear.

### 3.2 Mechanical Distribution of Dentition and Alveolar Bone

The maximum equivalent stress of the dentition in the four models is concentrated on the neck region of the anchoring tooth. There are differences in the force of the dentition with different expanders, and MSE can reduce the stress concentration on the neck of the anchored teeth. Otherwise, the shape of the palate has a great influence on the force of the dentition. It can be found that the force on the dentition of the model with a high arch of the palate is reduced compared with a normal arch, but their mechanical distribution on the dentition is similar.

**Figure 8:**
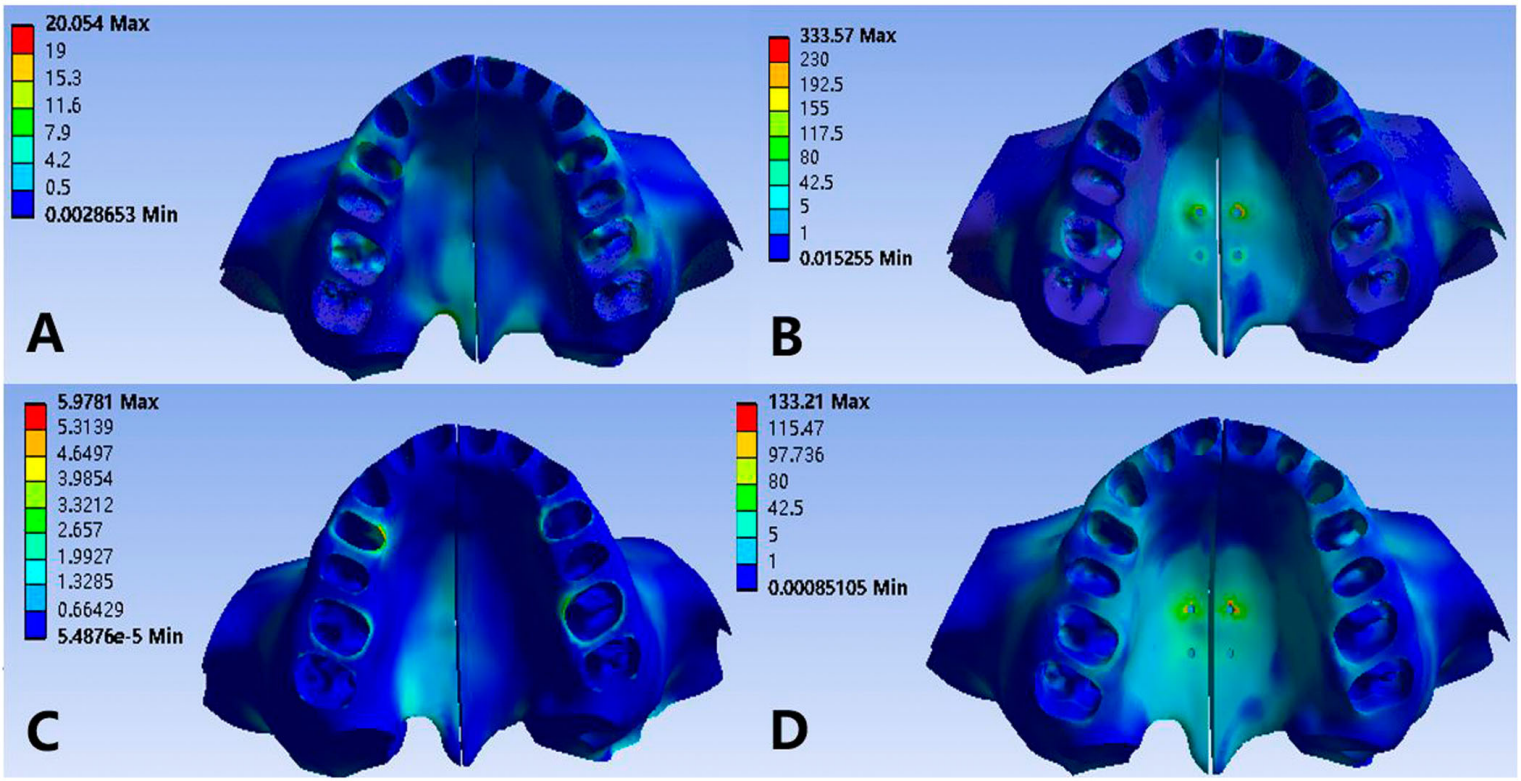
Von Mises stress comparison on alveolar bone. (A) Normal palate + RME, (B) normal palate + MSE, (C) high palate + RME, and (D) high palate + MSE.

As shown in Figure 8, the equivalent stress of the alveolar bone in Model 1 and Model 3 is mainly concentrated on the buccal and lingual bone wall of the anchoring tooth, while that in Model 2 and Model 4 is effectively reduced. In addition, no matter RME or MSE, the force of the alveolar bone in the high‐arch palatal group was lower than that in the normal palatal‐arch group.

### 3.3 Maximum Principal Stresses on Sutures

**Figure 9:**
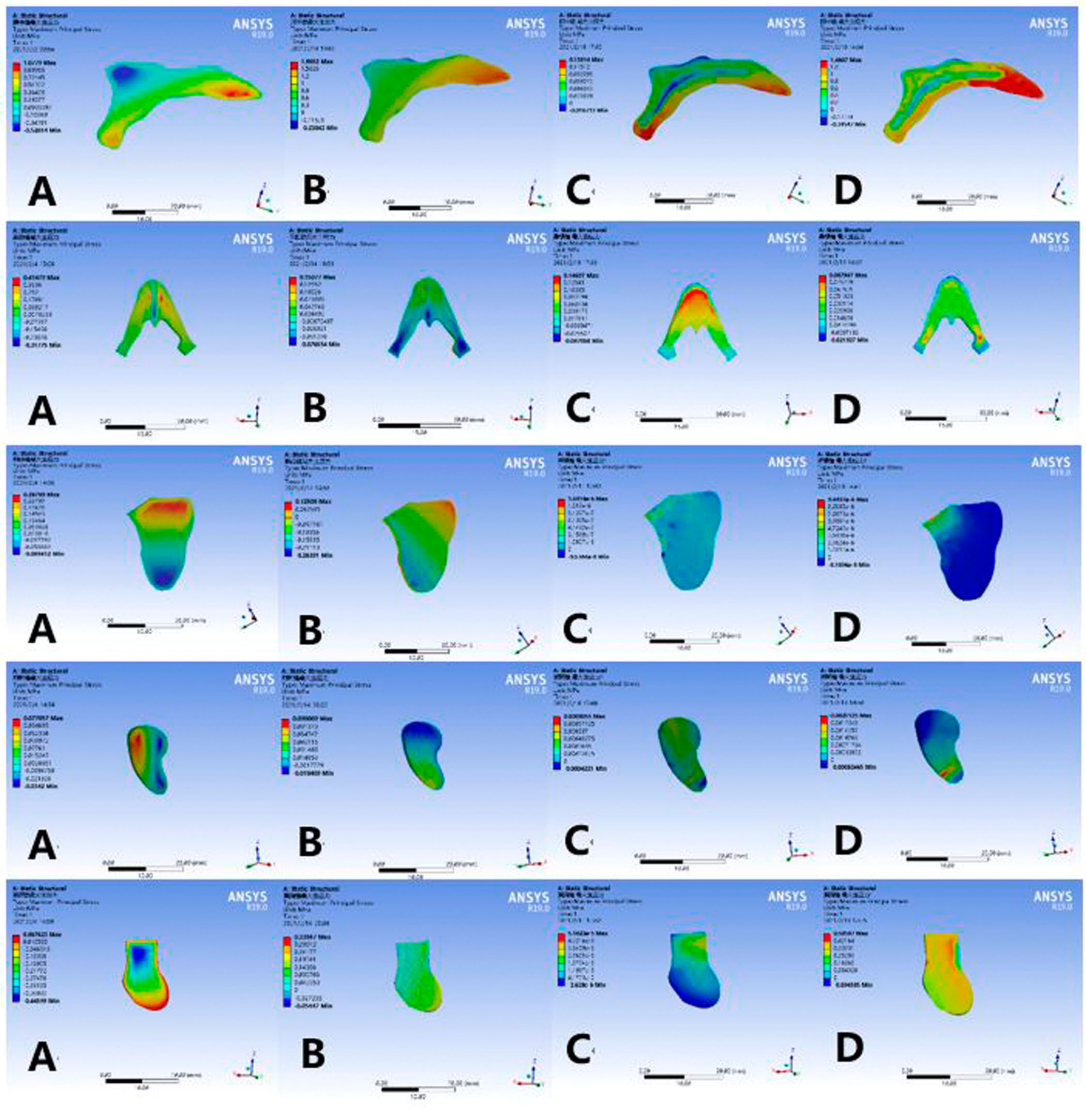
Maximum principal stresses on sutures (mid‐palatal suture, frontomaxillary suture, zygomatico‐maxillary suture, zygomaticotemporal suture, pterygopalatal suture). (A) Normal palate + RME, (B) normal palate + MSE, (C) high palate + RME, and (D) high palate + MSE.

As shown in Figure 9, from top to bottom the sutures are, in order, mid‐palatal suture, frontomaxillary suture, zygomaticomaxillary suture, zygomaticotemporal suture, and pterygopalatal suture. There are differences in the maximum principal stress of each suture in the four models and the maximum principal stress of MSE expansion on the medial palatine suture and pterygopalatine suture is much larger than that of RME expansion (Table 3).

**Table 3:** Comparison of maximum principal stresses on sutures.

	Model 1	Model 2	Model 3	Model 4
Frontomaxillary suture	0.10	−0.01	0.07	0.03
Zygomaticomaxillary suture	0.06	−0.05	0.00	0.00
Zygomaticotemporal suture	0.01	0.03	0.00	0.00
Pterygopalatine suture	−0.11	0.10	0.00	0.32
Mid‐palatal suture	0.16	0.83	0.06	0.81

### 3.4 Transverse Displacements of Palatal Sutures

**Figure 10:**
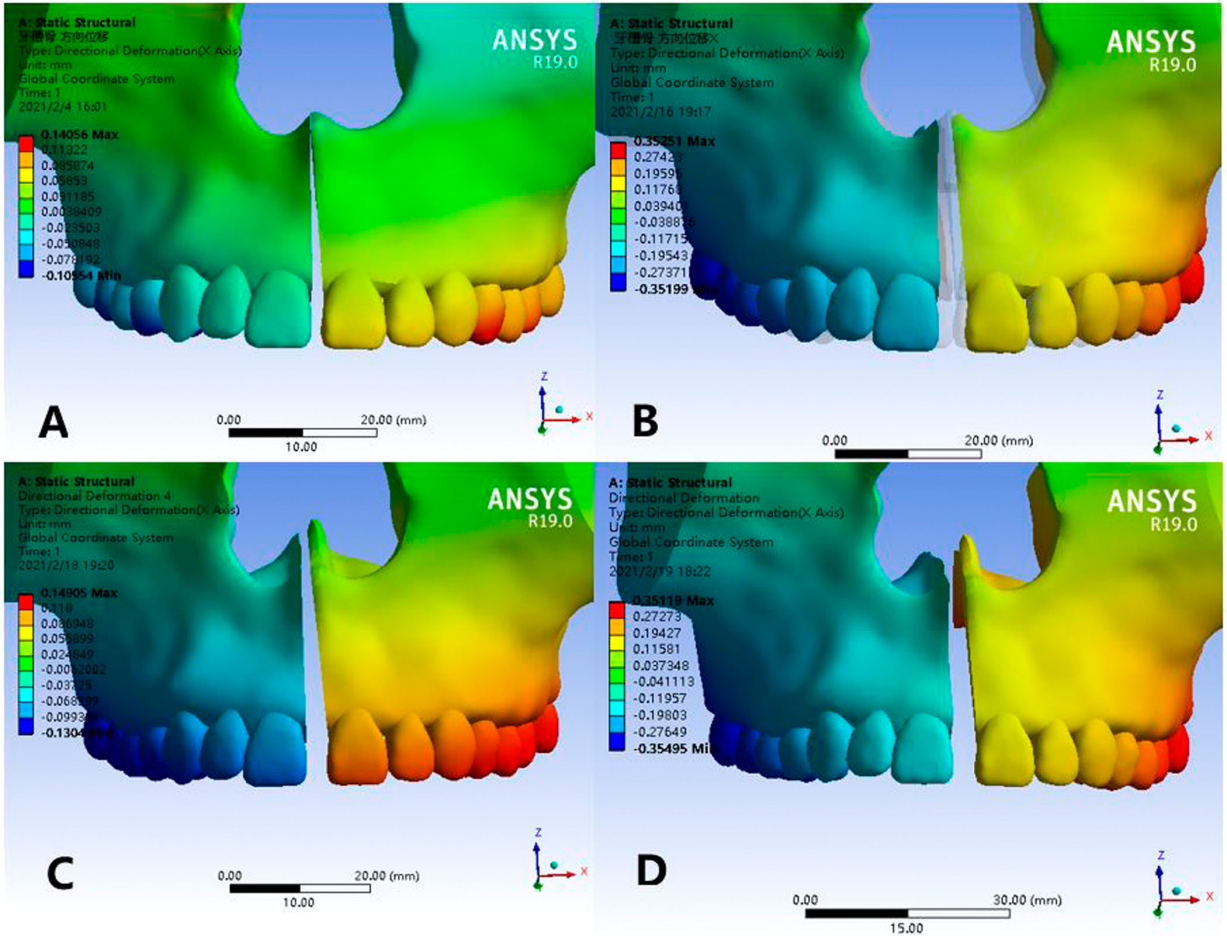
Transverse displacements of palatal sutures. (A) Normal palate + RME, (B) normal palate + MSE, (C) high palate + RME, and (D) high palate + MSE.

The lateral displacement of the palatal suture in Model 2 and Model 4 is significantly larger than that in Model 1 and Model 3. The mid‐palatal sutures of Model 1 and Model 3 are expanded in a “V” shape that is relatively broad at the anterior part and narrow at the posterior part, and the expansion of the mid‐palatal suture of Model 3 is greater than that of Model 1. However, the expansion of the anterior and posterior parts in Model 2 and Model 4 is basically the same, and the posterior part is slightly larger than the anterior part (Table 4). From the frontal view (Figure 10), it can be seen that the expansion of the upper and lower palatal sutures of Model 2 and Model 4 is basically the same, while the expansion of the upper part of the palatal suture in Model 1 and Model 3 is significantly smaller than that of the lower part, which is a V‐shaped pattern that is narrow in the upper part and wide in the lower part.

**Table 4:** Lateral displacement of palatal suture.

	Model 1	Model 2	Model 3	Model 4
P1	0.068526	0.1818	0.1048	0.19149
P2	0.053727	0.17598	0.089528	0.19241
P3	0.042791	0.18601	0.071418	0.19094
P4	0.042303	0.21763	0.064043	0.20882
P5	0.043846	0.23987	0.064442	0.23749

### 3.5 Comparison of Displacements of Maxillary

**Figure 11:**
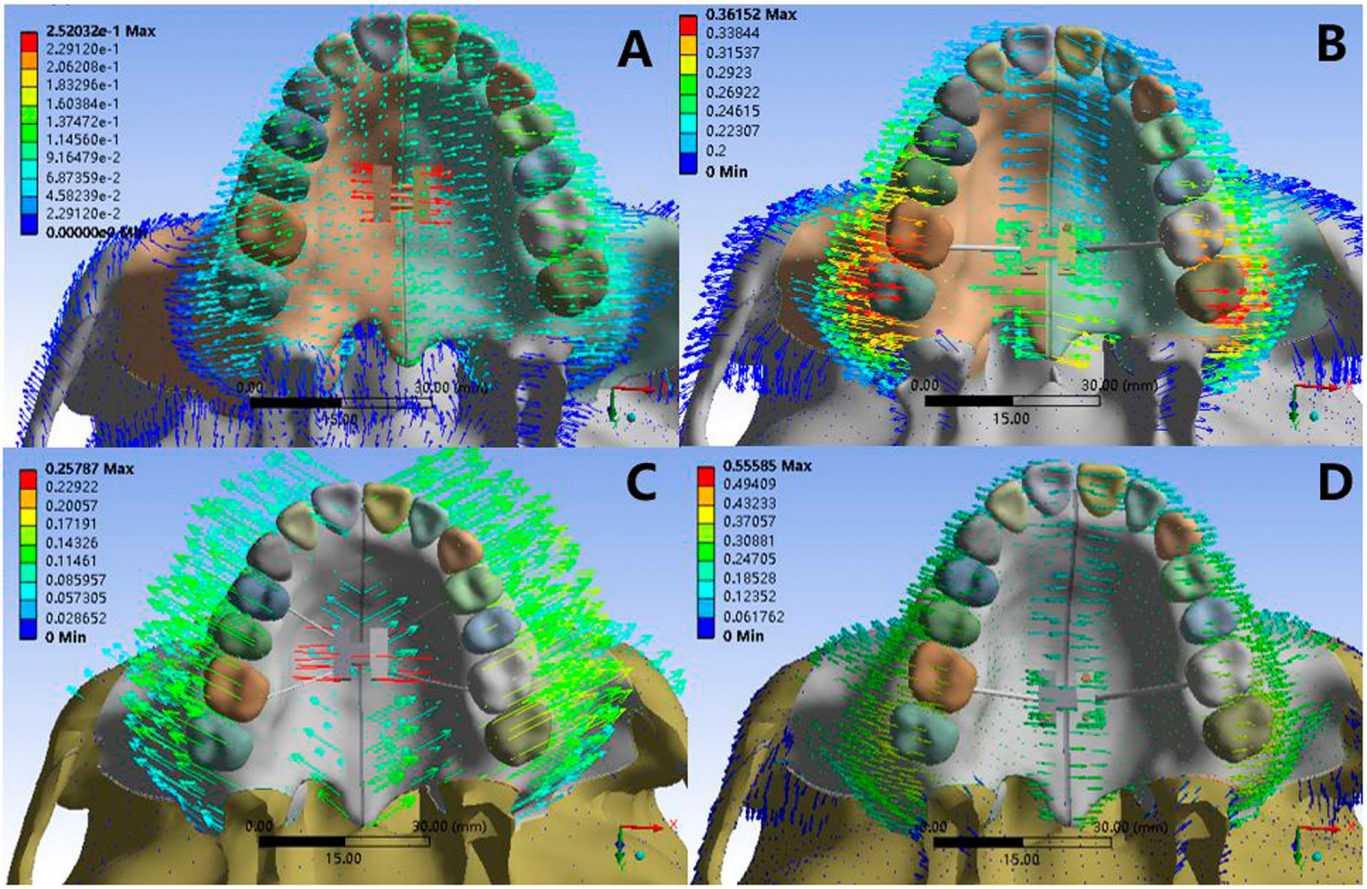
Comparison of displacements of maxillary (occlusal view). (A) Normal palate + RME, (B) normal palate + MSE, (C) high palate + RME, and (D) high palate + MSE.

**Figure 12:**
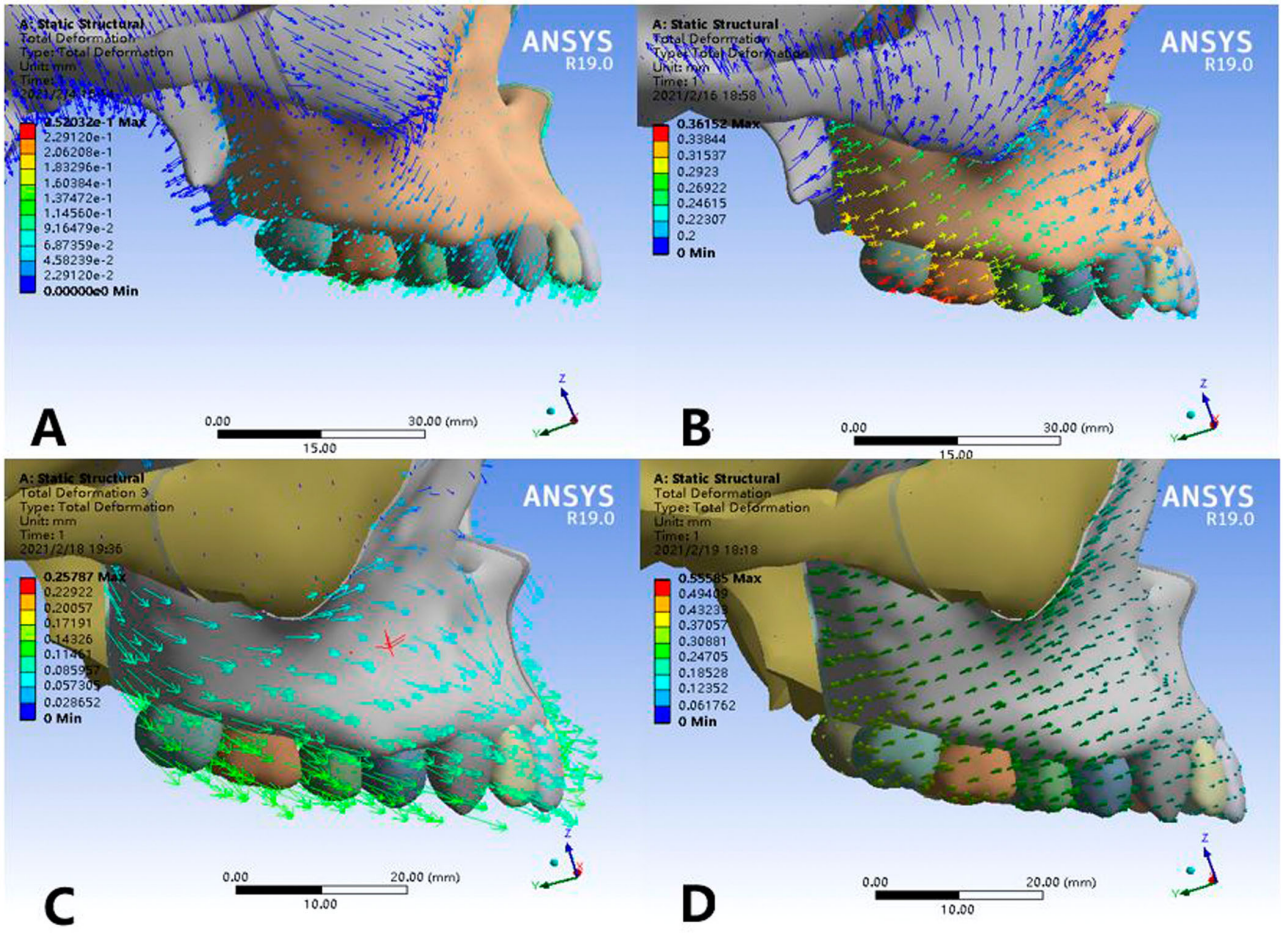
Comparison of displacements of maxillary (lateral view). (A) Normal palate + RME, (B) normal palate + MSE, (C) high palate + RME, and (D) high palate + MSE.

There were larger lateral displacements in all four models, which in Model 2 and Model 4 were significantly larger than those in Model 1 and Model 3. In the sagittal direction, the displacement of the maxillary in the four models is small, but there are differences in the displacement trends. In Model 1, the sagittal displacement trend of maxillary dentition is slightly backward while that of maxillary zygomatic process is forward. In Model 2, the upper incisors with its alveolar bone move a little backwards, and other parts of the maxillary complex have a forward movement in the sagittal direction. The maxilla in Model 3 shows forward movement in the sagittal direction. The Model 4 has no obvious displacement in the sagittal direction. Compared with the other three groups, Model 3 has a more obvious forward‐outward rotation trend (Figures 11 and 12).

### 3.6 Three‐Dimensional Displacement of Dentition

The amount of three‐dimensional displacement of dentition is shown in Table 5.

**Table 5:** Three‐dimensional displacement of dentition.

	Model 1			Model 2			Model 3			Model 4		
	X	Y	Z	X	Y	Z	X	Y	Z	X	Y	Z
Crown												
U1	0.082	0.040	−0.057	0.120	−0.051	−0.045	0.173	0.028	−0.009	0.177	0.005	−0.001
U2	0.086	0.024	−0.048	0.124	−0.065	−0.030	0.190	−0.009	−0.002	0.192	−0.036	0.001
U3	0.094	0.010	−0.034	0.130	−0.075	−0.023	0.215	0.008	0.015	0.221	−0.043	0.013
U4	0.137	0.016	−0.020	0.137	−0.080	−0.022	0.249	−0.021	0.028	0.249	−0.070	0.021
U5	0.110	−0.000	−0.015	0.140	−0.082	−0.023	0.275	−0.041	0.040	0.279	−0.092	0.028
U6b	0.118	0.004	−0.008	0.143	−0.080	−0.025	0.301	−0.052	0.047	0.301	−0.105	0.031
U6p	0.128	0.030	−0.031	0.147	−0.066	−0.050	0.321	−0.013	0.024	0.315	−0.073	0.017
U7b	0.103	−0.006	0.001	0.141	−0.078	−0.028	0.324	−0.075	0.058	0.328	−0.124	0.037
U7p	0.106	0.006	−0.020	0.145	−0.065	−0.050	0.346	−0.031	0.036	0.345	−0.090	0.022
Root												
U1	0.034	0.042	−0.060	0.074	−0.023	−0.047	0.134	0.046	−0.022	0.158	0.021	−0.005
U2	0.041	0.032	−0.054	0.083	−0.035	−0.036	0.150	0.023	−0.011	0.175	−0.003	0.002
U3	0.025	0.025	−0.038	0.065	−0.032	−0.026	0.139	0.017	0.007	0.170	−0.039	0.008
U4	0.035	0.002	−0.017	0.084	−0.054	−0.014	0.179	−0.028	0.030	0.198	−0.082	0.023
U5	0.047	0.010	−0.019	0.088	−0.052	−0.020	0.201	−0.033	0.034	0.221	−0.083	0.024
U6b	0.045	0.004	−0.011	0.0861	−0.054	−0.015	0.211	−0.050	0.044	0.232	−0.098	0.031
U6p	0.053	0.018	−0.035	0.094	−0.040	−0.044	0.240	−0.005	0.022	0.255	−0.059	0.015
U7b	0.052	−0.001	−0.002	0.094	−0.055	−0.018	0.245	−0.062	0.053	0.259	−0.110	0.037
U7p	0.055	0.011	−0.029	0.097	−0.043	−0.041	0.260	−0.028	0.032	0.27404	−0.076254	0.020

**Figure 13:**
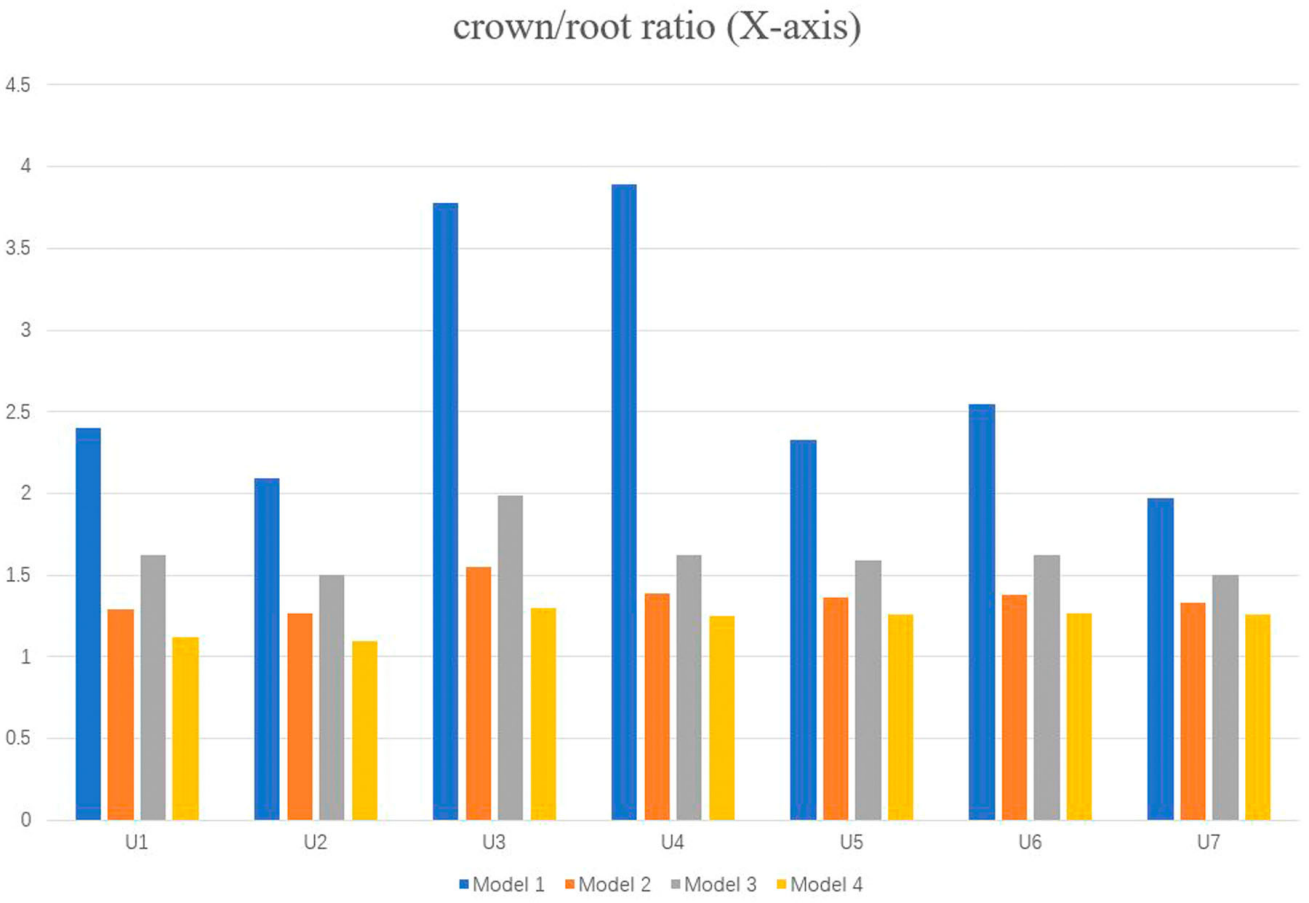
Comparison of crown‐to‐root ratio in the *X*‐axis direction.

In the *X*‐axis direction, the displacement trends of the crown and root in each model are consistent. The movement of the crown and root landmarks in Model 2 and Model 4 is significantly larger than that in Model 1 and Model 3. The displacements of crowns and roots in Model 3 are larger than those in Model 1, and the difference in root displacement is greater. The crown‐to‐root ratio in the *X*‐axis direction in Model 1 is significantly larger than that in the other three groups, and the crown‐to‐root ratio in Model 4 is the smallest (Figure 13).

The sagittal displacement in Model 3 and Model 4 is larger than that in Model 1 and Model 2, and the direction is basically along the negative direction of the *Y*‐axis.

In the *Z*‐axis direction, the moving directions of the crowns and roots in Model 1 and Model 3 are both negative directions, which means tooth extrusion. However, the crowns and roots of the posterior teeth in Model 2 and Model 4 moved along the positive direction of the *Z*‐axis, showing the intrusion of the teeth. The intrusion of the posterior teeth in Model 4 is greater than that of Model 2.

## 4 Discussion

The growth and morphology of the palate are influenced by genetic and environmental factors and are determined by multiple factors, including timing of development, soft tissue, and peak growth periods. There is a close relationship between the shape of the palate, the body of the tongue, and the shape of the dental arch (Hashimoto et al. 2014; Kurabeishi et al. 2018; Yu and Gao 2019), indicating that the shape of the palate is of great significance for clinical diagnosis and treatment. MTD is one of the most common malocclusions for which RME and MSE are usual treatments, but their clinical effects are affected by several factors, one of which is palatal shape. In clinical practice, many adolescent patients with mouth breathing have a narrow‐high palate. Meanwhile, skeletal class Ⅲ patients with maxillary underdevelopment and normal palatal morphology are also common in the clinic. They all need to be treated with maxillary expansion. However, the width and height of the palate may have an impact on the mechanical distribution and effect of the maxillary expansion. Consequently, it is necessary to explore the influence of palatal morphology on the treatment of arch expansion and the PI is used to objectively reflect the relative palatal height and evaluate the palatal morphology.

At present, Matsuyama et al. (2015) studied the effect of the depth of the palate on the maxillary expansion and established maxillary models with the depth of the palate increased by 4 and 8 mm, respectively. They only established the maxilla model but not the craniomaxillary complex model and found that the model with a high vault has the smallest lateral displacement of teeth, the expansion of the mid‐palatal suture, and the largest deformation of the expanding arm. The results of this study are different, and we found that the lateral displacement of the palatal suture in the high‐arched palatal group was larger during RME expansion, which may be related to the larger stress concentration in the upper part of the craniomaxillary complex and greater rotation of the maxillary body because of its center of resistance moving up. In addition, Its extrusion of the posterior teeth is more obvious. Therefore, the vertical control of posterior teeth should be paid attention to when performing RME expansion in patients with high palatal arch.

Previous studies have found that the vertical height of the Hyrax expander will affect the tipping movement of the anchoring teeth. When the vertical height of the expander is level with the center of resistance of the anchoring teeth, the tipping movement of the anchoring teeth is very small. Conversely, tipping movement of the anchoring teeth will occur (Araugio et al. 2013; Gómez‐Gómez et al. 2019). The shape of the palate will affect the placement position of the expander, especially the vertical position, which may be an important reason for the different stress distribution and displacement trends of patients with different palate shapes during expansion. These still need to establish more craniomaxillary models with different palatal morphology and expanders for further research to more accurately guide the placement and application of the expander in the clinic. In this study, we found that the high‐palate group has greater palatal suture widening and less tipping movement of anchor teeth, while the normal‐palate group has more pronounced tooth inclination movement, so we should pay attention to the side effects caused by the tipping movement of the teeth when treating a patient with normal palate. Some works of literature have shown that the use of micro‐implants to assist maxilla expansion can effectively expand the palatal base bone and improve the effectiveness of the expansion (Lee et al. 2010; Lee, Moon, and Hong 2017). The results of this study found that the shape of the palate had little effect on the expansion effect of MSE, and MSE can achieve greater palatal suture expansion and vertical control and avoid adverse dental effects.

Recently, the research on the mechanical effect of maxillary arch expansion is still controversial. This study found that there are stress concentrations around the piriform foramen, the frontal process of the maxilla, the medial orbital wall, and the orbital floor during RME. Not only does the resistance of the midpalatal suture need to be overcome, but also the maxilla and surrounding bone tissue are the sources of resistance to arch expansion, which is consistent with other research results (MacGinnis et al. 2014). In addition, this study found that the stress in the MSE group was mainly concentrated in the palatal bone, especially the area around the implants, and the two anterior implants were more stressed. Therefore, attention should be paid to prevent the loosening of the anterior implants due to stress concentration. Additionally, stress distribution was also observed around the medial orbital wall, zygomatic bone, and nasal bone, indicating that it also had mechanical effects on craniofacial bone tissue. However, Nelson Elias et al. (2017) showed that the palatal bone was subjected to the greatest tension, and the sphenoid pterygoid process was subjected to the greatest pressure.

There are still controversies between RME and MSE on the effect of bone sutures. Leonardi et al. (2011) found that most of the sutures around the maxilla were affected by RME. Ghoneima et al. (2011) believed that the force generated by RME mainly affected the anterior sutures of the craniomaxilla, such as the mid‐palatine suture and front‐maxillary sutures, rather than the posterior sutures, such as the zygomatic‐maxillary sutures. Our study also found that the equivalent stress of the frontonasal suture and the mid‐palatal suture during RME was greater than that of the posterior sutures, which may lead to V‐shaped expansion of the mid‐palatal suture. Some studies have reported that the resistance of the posterior part of the palatal bone increases with age (Melsen and Melsen 1982; Lee et al. 2021). The maximum principal stress and equivalent stress of the pterygopalatine suture in the MSE group were greater than those in the RME group, indicating that MSE could better overcome the resistance of the posterior part of the palatine bone, which may be the reason why MSE allows parallel expansion of the mid‐palatal suture and can be applied to adult patients.

In this study, finite element analysis was used to study the effect of RME and MSE on the craniomaxillary complex with different palatal morphology. The RME and MSE models were established according to the actual clinical size of the expander, and reasonable material properties were defined for the craniomaxillary structures by consulting a large amount of literature, which can be closer to the actual clinical situation and provides valuable guidance for the selection, placement, and program design of the expander for patients with different palate shapes in the clinic.

The limitation of this study is that finite element analysis can only simulate bone tissue and transient effects but not long‐term changes, so the development of finite element is needed to best simulate the real effects of maxillary expansion. In the future, it is possible to try to establish a finite element model with soft and hard tissues of the craniomaxillofacial region under continuous growth to better simulate the clinical practice of maxillary expansion.

## 5 Conclusion

The simulations of the mechanical effects of RME and MSE on the maxillofacial complex under different palatal shapes with the finite element analysis show the following.

1. Different shapes of the palate interfere with the effects of RME and MSE, and its influence on the stress distribution and displacement of the craniomaxillary complex when using RME is higher than MSE.
2. The lateral displacement of the palatal suture of MSE is significantly larger than that of RME. The mid‐palatine suture in RME expansion is widened in a V‐shape while that in the MSE expansion is evenly expanded, which may be related to the ability that MSE better overcome the resistance of the posterior part of the craniomaxillary complex.
3. It is more prone to tipping movement of the anchor teeth using RME under normal palate, and MSE may manage the vertical control better due to the smaller crown/root ratio than RME and intrusive movement of molars.

## Author Contributions

Yaohui Pan contributed to the conception and design of this study, performed the main literature retrieval, data acquisition, and statistical analysis, and wrote and revised the manuscript. Wenjing Peng helped perform the literature retrieval, graphic processing, and statistical analysis and drafted the manuscript. Yanyu Wang performed the statistical analysis and helped a lot with the major revision.

## Conflicts of Interest

The authors declare no conflicts of interest.

## Data Availability

The data that support the findings of this study are available from the corresponding author upon reasonable request.
